# Hydration of Hybrid Alkaline Cement Containing a Very Large Proportion of Fly Ash: A Descriptive Model

**DOI:** 10.3390/ma9070605

**Published:** 2016-07-22

**Authors:** Inés Garcia-Lodeiro, Shane Donatello, Ana Fernández-Jiménez, Ángel Palomo

**Affiliations:** Cement and Recycling Materials Department, Eduardo Torroja Institute (IETcc-CSIC), Madrid 28033, Spain; shane.donatello@ec.europa.eu (S.D.); anafj@ietcc.csic.es (A.F.-J.); palomo@ietcc.csic.es (Á.P.)

**Keywords:** hybrid alkaline cement, alkaline activation, fly ash, geopolymer, descriptive hydration model, gel microstructure

## Abstract

In hybrid alkaline fly ash cements, a new generation of binders, hydration, is characterized by features found in both ordinary portland cement (OPC) hydration and the alkali activation of fly ash (AAFA). Hybrid alkaline fly ash cements typically have a high fly ash (70 wt % to 80 wt %) and low clinker (20 wt % to 30 wt %) content. The clinker component favors curing at ambient temperature. A hydration mechanism is proposed based on the authors’ research on these hybrid binders over the last five years. The mechanisms for OPC hydration and FA alkaline activation are summarized by way of reference. In hybrid systems, fly ash activity is visible at very early ages, when two types of gel are formed: C–S–H from the OPC and N–A–S–H from the fly ash. In their mutual presence, these gels tend to evolve, respectively, into C–A–S–H and (N,C)–A–S–H. The use of activators with different degrees of alkalinity has a direct impact on reaction kinetics but does not modify the main final products, a mixture of C–A–S–H and (N,C)–A–S–H gels. The proportion of each gel in the mix does, however, depend on the alkalinity generated in the medium.

## 1. Introduction

Modern construction is unthinkable without ordinary portland cement (OPC), the fundamental binder in concrete. According to CEMBUREAU data, some four billion tons of PC were manufactured worldwide in 2013 alone. With the use of fossil fuels to raise kiln sintering temperatures to around 1450 °C and the limestone decarbonation required to produce raw meal, the industry accounts for approximately 5%–8% of anthropogenic CO_2_ emissions [1].

Attempts to reduce its CO_2_ footprint by blending OPC with supplementary cementitious materials (SCMs), such as coal fly ash, metakaolin or blast furnace slag [2], have yielded pozzolanic cements with technical properties comparable to those of ordinary cement [3]. Nonetheless, such blended cements contain 70 wt % to 90 wt % OPC. On the opposite end of the spectrum are alkali-activated binders containing 0 wt % OPC. There the reactive solid is an aluminosilicate material prone to dissolution under highly alkaline conditions. Given the important role of aluminosilicate phases, generally speaking materials that hold promise as alkali-activated cements also exhibit pozzolanic activity in blended cements: i.e., coal fly ash [4,5] and metakaolin [6]. The hydration of these alkali-activated cements, also termed geopolymers, which has been studied in considerable detail over the last 10–15 years, has been shown to be governed by mechanisms that are different to OPC. The major difference between the two is that alkali activated cements are mixed not with water but highly alkaline chemicals [7,8,9]. In addition, in the case of fly ash, a moderately high curing temperature (60 °C to 90 °C) is required to ensure practical reaction kinetics [10].

Fly ash is the supplementary cementitious material most widely used, ton for ton. Taking OPC and alkali-activated fly ash (AAFA) as the two extremes on a spectrum, blended cements containing fly ash (pozzolanic cements) would represent the middle ground. The ceiling fly ash dry mass content in pozzolanic binders is usually set at 20% to 55% [11]. Because the pozzolanic reaction of fly ash is fairly slow, early age strength has been shown to be lower and setting times longer in cements with a high FA content than in other pozzolanic binders [12]. Attempts made to surmount these drawbacks include the use of quick-setting cement [13] or the addition of limestone to the mix [14,15].

**Figure 1:**
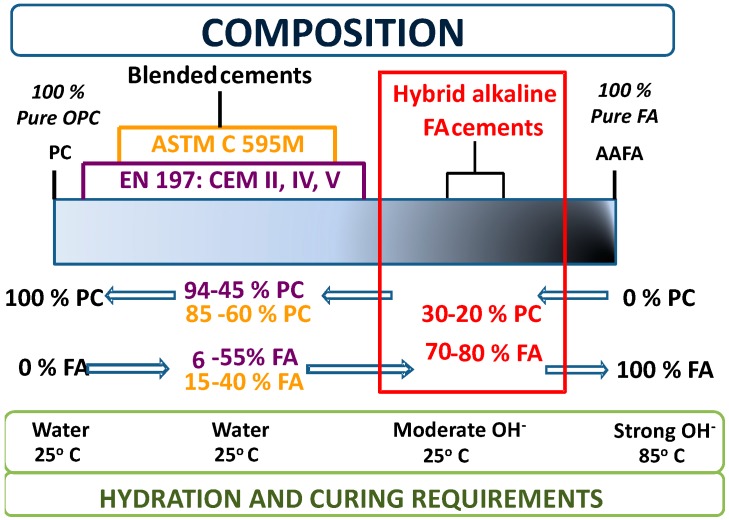
Position of hybrid alkaline fly ash cements on the pure Portland cement (PC)-pure alkali activation of fly ash (AAFA) spectrum, relative to pozzolanic fly ash cements.

Alternatively, a suitable alkali may be used to enhance fly ash reactivity [16,17]. Research is underway at this time on what are known as ‘hybrid alkaline fly ash cements’ [18,19,20,21,22,23,24,25,26], typically binders with a low Portland cement and a fly ash content significantly higher than in blended pozzolanic cements. The source of the alkali may be a highly alkaline solution, used in lieu of mixing water, a solid or a dissolved Na/K compound. In all three cases, the hybrid alkaline cements react and can be cured at ambient temperature. These cements can also be regarded to lie in an intermediate position on the OPC/alkali activation of fly ash (AAFA) spectrum, as illustrated in Figure 1.

While much research has been conducted on the mechanisms governing hydration in OPC [27,28,29], pozzolanic [30,31] and AAFA [32,33,34] cements, very little has been published on the hydration mechanisms in hybrid alkaline systems

This paper aims to describe the hydration mechanisms taking place in fly ash-high hybrid alkaline cements and define the main characteristics of the cementitious gel forming in those systems. Given the significant role of the nature of the alkaline admixture in such cements, it is also discussed hereunder.

## 2. Background

Inasmuch as hybrid alkaline cements may be regarded to lie somewhere between the two extremes defined by OPC and AAFA, a brief discussion of the hydration mechanisms present in the latter two systems is in order, applying the same methodology as subsequently used to describe the model proposed for hybrid cements.

### 2.1. Portland Cement (PC) Hydration

**Figure 2:**
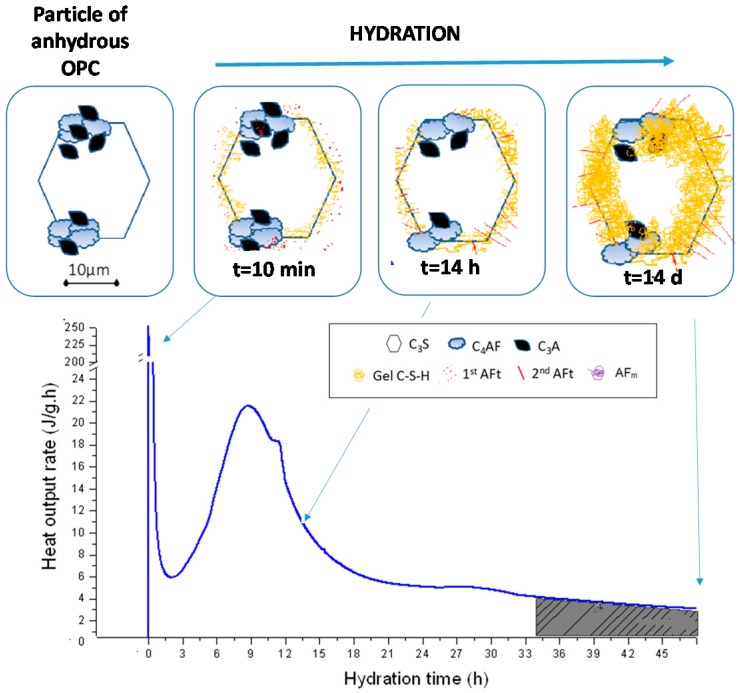
Descriptive model for poly-phase OPC grain hydration and a typical calorimetric curve for OPC hydration (grain drawings adapted from Scrivener as quoted in Taylor (1997) [27]).

Portland cement clinker consists primarily in four reactive phases: Alite (C_3_S), belite (C_2_S), tricalcium aluminate (C_3_A) and ferrite (C_4_AF). The most reactive of these components are C_3_A [35,36] and C_3_S [37,38,39]. A single grain of cement may be divided into several regions containing different clinker phases. Figure 2 shows a widely accepted descriptive model [40] (first proposed by Scrivener in 1984) [41]. Figure 2, also reproduces a typical calorimetric analysis of OPC hydration for comparison.

Further to Figure 2, a very intense but short-lived exothermal peak generated by the rapid dissolution of the surfaces of C_3_S and C_3_A concurs with the initial wetting of cement grains. Clinker dissolution raises the liquid phase pH substantially, along with Ca^2+^, Al^3+^ and silicate ion concentrations. The sulfate present in the gypsum induces a decline in Al^3+^ concentration in the liquid phase due to the precipitation of tiny ettringite (AFt) prisms. C_3_A hydration is subsequently inhibited by the presence of sulfate ions. The reason advanced is that the AFt forms layers on the surface of the C_3_A grains, making it inaccessible to the liquid phase [40]. Early age precipitation of C–S–H or C–(A)–S–H gels on the surface of clinker grains also lowers effective clinker solubility and with it the rate of heat released to a characteristic minimum during the induction period.

While continuing at a much slower rate, clinker hydration does not stop altogether during the induction period. Ca and silicate ion concentration in the liquid phase gradually rises until large amounts of C–S–H gel precipitate, removing the Ca and silicate ions from the solution and favouring the resumption of C_3_S phase hydration [42,43]. That leads to the formation of large amounts of so-called ‘outer’ C–S–H gel, with the coalescence of cement particles to which cement paste setting is attributed [44]. This period of massive gel precipitation, termed the acceleration period, constitutes the predominant exothermal event in PC hydration after initial wetting.

As sulfate ion activity declines in the liquid phase, the C_3_A grain surfaces come into direct contact with the liquid and the reaction resumes, forming larger ‘secondary ettringite’ prisms. The resumption of C_3_A hydration is often visible in calorimetric data as a shoulder on the downward slope of the acceleration stage, although at times it is undistinguishable from the main acceleration/deceleration peak. ‘Inner’ C–S–H gel also gradually forms in this period.

As the amount of inner C–S–H gel rises, lowering sulfate availability, monosulfate (AFm) is favored over trisulfate (AFt) formation. Although the mechanism has yet to be accurately described, the larger AFt prisms appear to be unaffected, since as a rule the lower relative surface area renders larger crystals more stable than smaller ones [45,46]. The conversion of AFt to AFm or the reaction between C_3_A and AFt to form AFm is sometimes visible in the form of a low intensity, wide exothermal peak appearing between days 1 and 3. The fairly small amount of heat that continues to be generated even after three days can be attributed to the ongoing hydration of C_3_S grains, with the formation of more inner C–S–H gel. That process is related to the significant gains in compressive strength observed in PC pastes after the second day.

The nanostructure of the resulting C–S–H gel consists of a central layer of octahedrally orientated Ca–O units sandwiched by upper and lower layers of imperfect silicate tetrahedral chains. These chains are formed from units consisting of three tetrahedra in which two are linked by a third, the ‘bridging’ tetrahedron. The predominance of Q^1^ (end of chain) and Q^2^ (mid-chain) units in ^29^Si MAS NMR spectra confirms that the silicate chains in C–S–H gel are weakly polymerized [27,47,48].

### 2.2. Alkali-Activated Fly Ash Cement Hydration

As noted earlier, AAFA and OPC hydration are wholly different processes. Fly ash reactivity is based on the alkaline dissolution of disordered aluminosilicate networks. A large fraction of fly ash particles is characterized by a peculiar morphology: spheres that may or may not house other smaller spheres. The material consists of a vitreous phase with a few minority crystals such as quartz (5% to 13%), mullite (8% to 14%), hematite/magnetite (3% to 10%) and, on occasion, corundum or lime [49,50].

**Figure 3:**
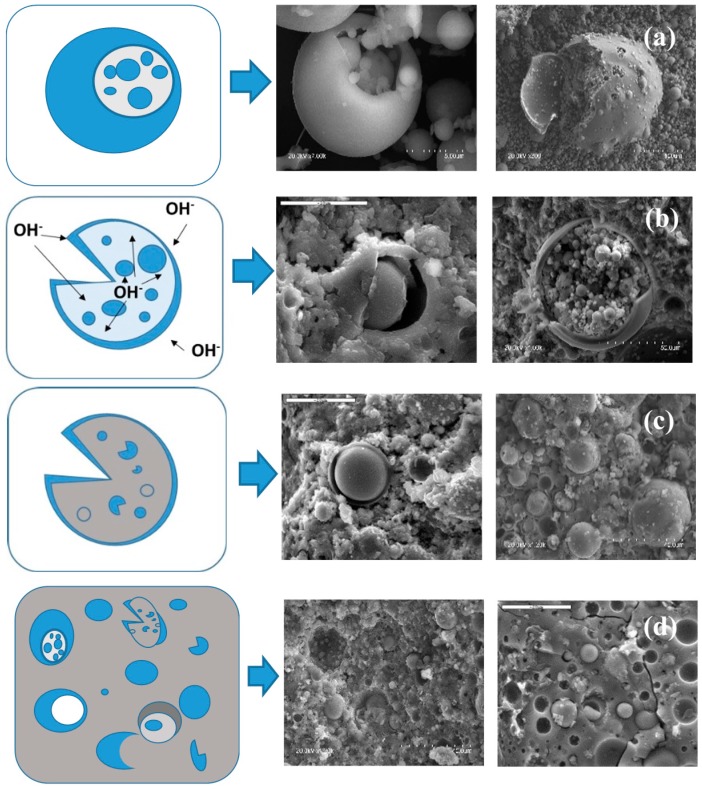
Conceptual model for AAFA cement hydration. (**a**) Starting material; (**b**) initial alkaline attack on the ashes and early N–A–S–H gel formation; (**c**) gel polymerisation and positioning on the inner and outer surfaces of the exposed fly ash; and (**d**) mature and heterogeneous AAFA cement paste microstructure (adapted from Fernández and Palomo [7]).

In 2005, Fernández and Palomo [7] proposed a conceptual model for the alkaline activation of fly ash (see Figure 3). The process would begin with a chemical attack on the ash surface, resulting in the formation of small cavities in the walls of the ash particles, exposing the tiny particles on the inside to the action of the alkalis. In this stage of the reaction the alkalis would attack from inside and outside the particles (Figure 3b). The ash would continue to dissolve and the reaction products generated inside and outside the ash crust would precipitate, covering the smaller unreacted spheres and hindering their contact with the alkaline solution (Figure 3c). Alkaline activation would ultimately continue slowly, for once the ash particles are covered by the reaction products, alkaline attack would take place via diffusion only. At the end of the process, a number of morphologies may co-exist in the same paste: Unreacted ash particles, particles under alkaline attack and reaction products (N–A–S–H gel or zeolites). This model is normally applied to the aforementioned ‘cenospheres’ (hollow particles or particles housing smaller spheres), which account for around 30% of the total. All the other particles, which are solid ‘plerospheres’ [51], exhibit greater or lesser reactivity depending on their size. The smallest particles (under one micrometre, approximately 10% of the total) dissolve rapidly in a highly alkaline medium. Despite these rather low percentages, the speedy setting and high early age strength attained with alkaline activation can be largely explained by the intense reactivity of cenospheres and small plerospheres. 

Glukhovsky [52] was the first to propose a general nano-scale mechanism for alkali activation reactions in aluminosilicate materials. His model is based essentially on dissolution-precipitation. The process begins when a series of silica and alumina monomers are released into the medium as a result of the rupture of Si–O–Si and Si–O–Al bonds attendant upon the dissolution of a source of aluminosilicate. The silica monomers inter-react to form dimers, which then react with other silica or alumina monomers to form trimers, and so on. As polymerization advances, a gel consisting in a complex aluminosilicate grid precipitates. The alkaline ion compensates the charge deficit stemming from the replacement of Si^4+^ with Al^3+^. In the very short term (minutes to hours), the gel formed has a fairly high Al content as a result of the high Al^3+^ ion concentration in the alkaline medium: reactive aluminum dissolves more quickly than reactive silicon because Al–O bonds are inherently weaker than Si–O bonds. As the reaction progresses, more Si–O groups dissolve, raising the silicon concentration in the reaction medium and enhancing its uptake in the gel. The N–A–S–H gel formed in hardened pastes is XRD amorphous and has been labeled as a zeolite precursor [7]. 

The structure of the N–A–S–H gel differs substantially from the C–S–H gel formed in OPC hydration. These N–A–S–H gels are characterized by a three-dimensional structure in which the Si is found in a variety of environments, with a predominance of Q^4^(3Al) and Q^4^(2Al) units. The silicate and aluminate groups are tetrahedrally coordinated and joined by oxygen bonds. The negative charge on tetrahedrally coordinated Al is offset by the presence of the alkaline cation provided by the activator used (typically Na^+^).

## 3. Hybrid Alkaline Cement Hydration

In recent studies, the authors have explored the potential for combining OPC and AAFA systems to form what might be called ‘hybrid alkaline cements’. In these systems, the starting solid comprises small percentages (20 wt % to 30 wt %) of OPC or OPC clinker and large proportions (70 wt % to 80 wt %) of fly ash. They also contain a separate source of alkali. The most significant differences between these systems and pozzolanic cements lie in the proportion of OPC, the prevalent solid component in the latter, and the alkaline addition in the former. The pozzolanic reaction involving fly ash particles is known to be slow in pozzolanic cements, while fly ash reactivity kinetics are much faster in hybrid alkaline cements (particularly as regards the cenospheres and the smallest plerospheres).

**Figure 4:**
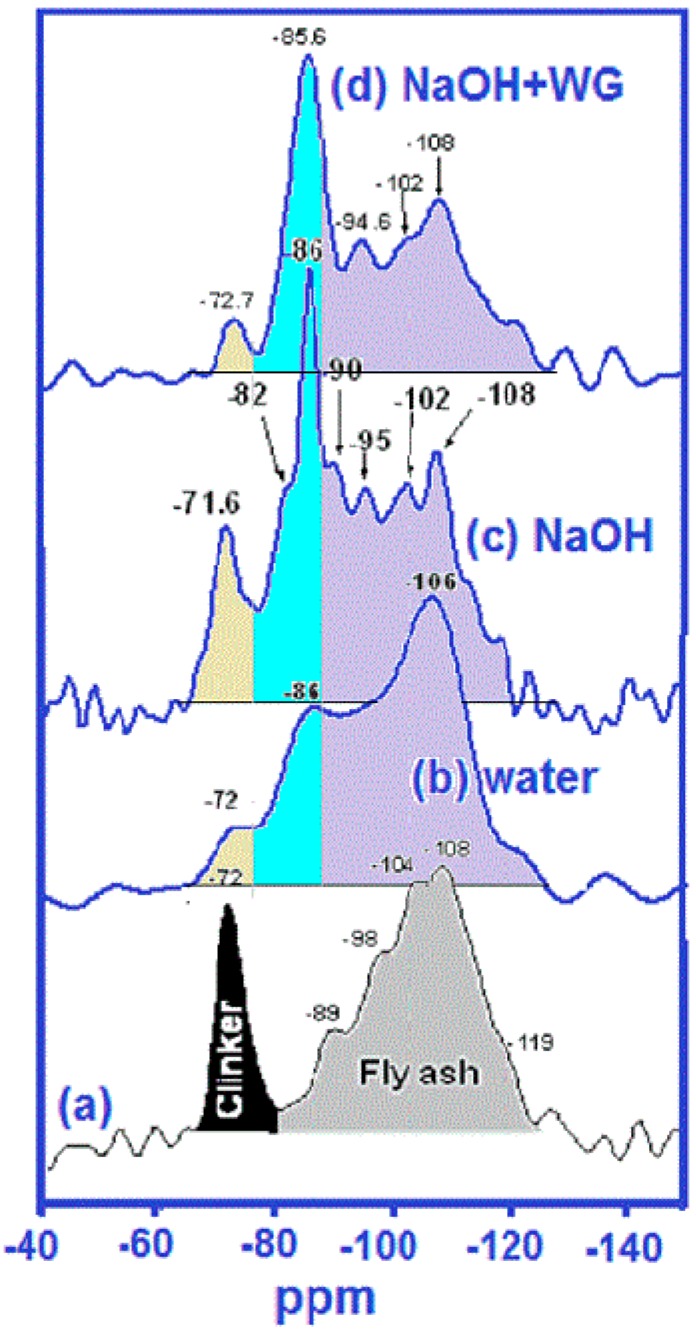
^29^Si MAS NMR spectra: (**a**) initial raw mix (30% clinker + 70% fly ash); (**b**) 28-day water-hydrated material; (**c**) 28-day NaOH-hydrated material; (**d**) 28-day NaOH + WG (waterglass)-hydrated material (adapted from Palomo et al. [18]).

In 2007, Palomo et al. [18] showed that according to the ^29^Si NMR findings for alkaline fly ash cement pastes, hydration in an NaOH medium significantly inhibited the calcium silicate clinker phase hydration and hence portlandite formation in the first 28 days. That notwithstanding, their 28-day compressive strength was similar to blended cement strength. FTIR spectra for the NaOH-hydrated paste exhibited several new absorption bands in the 960 cm^−1^ to 1050 cm^−1^ region, attributed to a combination of N–A–S–H– and C–S–H gels. That was supported by a predominant, clearly defined peak at −86 ppm on the ^29^Si NMR spectra for NaOH-activated hybrid cements (see Figure 4). Signals in that region may be indicative of Q^2^(0Al) environments typical of C–S–H gel [47,53,54] as well as Q^3^(3Al) and Q^4^(4Al) environments typical of N–A–S–H gels [32,55]. The intensification of the −86 ppm signal in NaOH-hydrated pastes was attributed to the additional Q^3^ or Q^4^ environments, or both, induced by the alkaline activation of fly ash particles.

The co-existence of these gels must be borne in mind when studying hybrid alkaline cements. Both N–A–S–H– and C–S–H gels, whose morphologies differ, have been observed in alkali-activated metakaolin/slag mixes [56]. The Ca/Si ratio in the C–S–H regions was around 1.0, a value significantly lower than found in the C–S–H gels forming in OPC hydration. In a later study on metakaolin/ground granulated blast furnace slag (MK/GGBFS), the aforementioned authors concluded that C–S–H gel stability declined when the activator was overly alkaline (>7.5 M NaOH). As a result, larger amounts of the Ca-containing component (GGBFS) were needed for the C–S–H regions to be detected [57]. Those findings were generally consistent with the conclusions of an earlier study on MK and Ca(OH)_2_ mixes by Alonso et al., in which C–S–H hydration was favored to some extent in a 5 M NaOH, but not in a 12 M NaOH medium [58,59].

Studies using synthetic gel phases have provided much valuable information [60,61,62]. Garcia-Lodeiro et al. [60] showed that the Ca/Si ratio in C–S–H gels declined in high alkaline media. Those authors also observed that when C–S–H gel was exposed to both an alkali and soluble Al, it evolved into a more highly polymerized C–A–S–H gel [62]. In studies on mixes of synthetic C–S–H and N–A–S–H gels, the same group reported that at pH > 12 the system ultimately tended toward the most stable product, C–A–S–H gel [63]. That tendency was recently confirmed in actual hybrid alkaline fly ash cements hydrated for up to one year [20].

**Figure 5:**
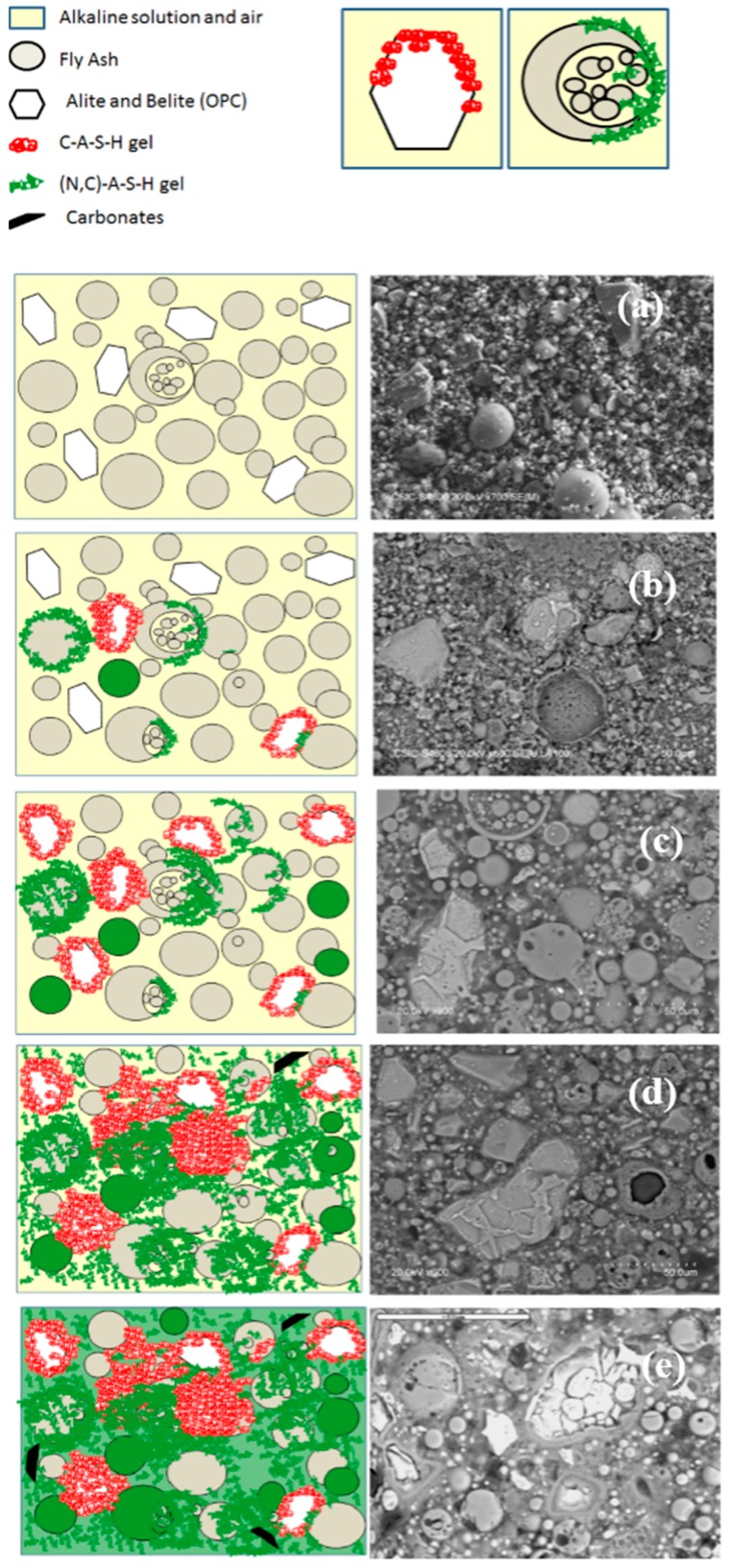
Changes in gel composition and microstructure of a hybrid alkaline cement with a very high fly ash content: (**a**) initial stage; (**b**) early age sample (min); (**c**) early age sample (h); (**d**) 7-day sample; (**e**) 28-day sample.

The BSEM (Backscattering electron microscopy) images of several hybrid alkaline cement samples [20,22,64,65,66,67] in Figure 5 illustrate this process. In the presence of the alkali (NaOH), substantial amounts of N–A–S–H gel, along with smaller quantities of C–S–H gel, would be expected to form at early ages. With the decline in liquid phase alkalinity as a result of both carbonation and the alkaline hydrolysis of vitreous fly ash phases, C–S–H gel stability rises. As significant amounts of soluble Al are released by the fly ash, however, C–A–S–H prevails over C–S–H formation. The generation of a pure N–A–S–H-like gel is unlikely in fly ash activation in hybrid alkaline cements, for the clinker present would release much more Ca than found in clinker-free pure AAFA cements. The outcome would be the partial replacement of the Na^+^ ions by Ca^2+^ ions as charge balancers, forming what might be labeled an (N,C)–A–S–H gel. Co-precipitation of the two types of gels has been confirmed in such systems [60,61,62,63]. 

The use of activators with different degrees of alkalinity has a direct impact on reaction kinetics. The addition of highly alkaline activators favors ash [67] over clinker [68,69] dissolution, whereas in moderately alkaline media clinker hydration is favored and ash dissolution retarded. With time, however, the main reaction products detected are the ones most thermodynamically stable, irrespective of the type of activator used. In hybrid systems with high ash and low OPC contents the result is a mix of C–A–S–H + (N,C)–A–S–H gels [20,22,64,65,66]. 

Garcia-Lodeiro et al. [22] used isothermal conduction calorimetry in an initial study on early age (72 h) reaction kinetics in a 100% OPC systems, 100% FA systems and in hybrid cement consisting of 30% OPC and 70% fly ash. To that end, they used two activating solutions: Na_2_CO_3_ and a mix of NaOH + Na_2_SiO_3_. Further to the data from several analytical techniques (BSEM, FTIR, DTA/TG, etc.), they concluded several things; (i) the presence of high alkali content-induced delays in normal Portland cement hydration; (ii) the presence of alkalis induced some degree of fly ash dissolution, this process is very slow at ambient temperature; (iii) Alkaline activators must be present to stimulate hybrid cement hydration. The hydration kinetics were substantially modified by the type of alkaline activator, particularly with respect to the secondary phases generated. The main reaction products, however, a mix of C–A–S–H and (N,C)–A–S–H gels, were unaffected by the activator. While the type of alkaline activator impacted reaction kinetics and the formation of secondary reaction products (carbonates, AFm phases, etc.) significantly, it did not appear to have any material effect on the main cementitious gels formed ((N,C)–A–S–H/C–A–S–H).

The thermodynamically stable majority product was a mix of cementitious gels that formed irrespective of the activator used. The use of Na_2_CO_3_ as an alkaline activator retarded gel precipitation, favoring the formation of secondary phases such as gaylussite and AFm-type species. Nonetheless, a larger proportion of gel phase appeared to precipitate than in the system activated with the solution containing NaOH + WG. Similar conclusions have been obtained by different authors [64,65,70] for alkali activated hybrid cements, but using solid activators [71].

**Figure 6:**
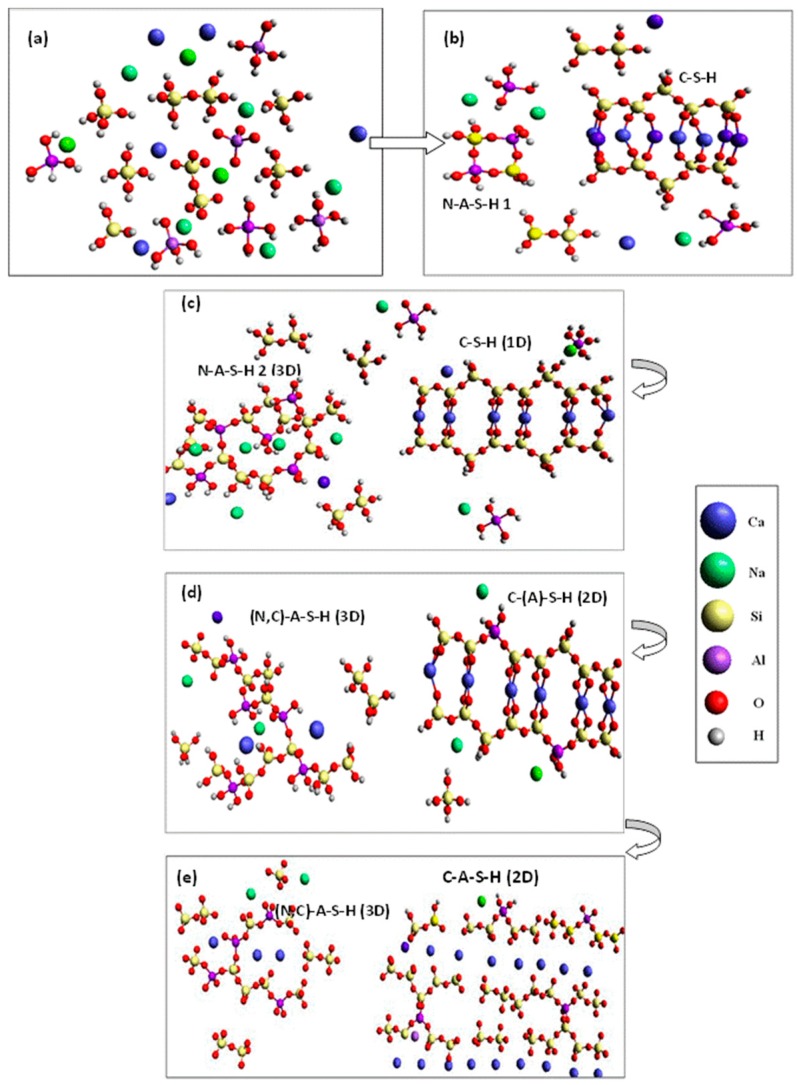
Nano-structural mechanism for gel formation in hybrid alkaline cements; (**a**) dissolution of ionic species from the source of alumino-and calcium silicates; (**b**) precipitation of aluminum-high (type I) N–A–S–H gels and C–S–H gels; (**c**) silica uptake by both gels with an increase in C–S–H gel mean chain length and the generation of silica-high type 2 N–A–S–H gels; (**d**) diffusion of aluminum and calcium in the matrix and their uptake, respectively, in C–S–H and N–A–S–H gels to form (N,C)–A–S–H gels; (**e**) distortion of the (N,C)–A–S–H gel due to the polarizing effect of calcium, leading to its rupture, while the C–A–S–H gel continues to take up aluminum species in bridging positions, favoring chain cross-linking and hence a more polymerized structure (hydrogen bonds omitted in this final stage).

^29^Si and ^27^Al NMR, together with TEM/EDX studies of hybrid alkaline cements hydrated for up to 1 year have shown that both C–S–H and N–A–S–H gels evolve toward cross-linked C–A–S–H gels. The authors found that the lack of sufficient total Ca in the paste prevented conversion of all the N–A–S–H to C–A–S–H gel [20]. The expected nano-structural gel evolution (based on prior studies [20]) is illustrated in Figure 6. The process begins with the dissolution of the source of silicoaluminates and calcium silicates in the alkaline solution, with the concomitant release of a wide variety of dissolved species (Figure 6a). The medium becomes saturated with ions that are not uniformly distributed but rather exhibit local concentrations of the various species, depending on the nature of the nearest particle [20].

When these local concentrations reach saturation, C–S–H and N–A–S–H gels precipitate simultaneously (competitive reactions), although which of the two precipitates more rapidly has yet to be determined (Figure 6b). As the reaction progresses, more Si–O groups dissolve out of the initial aluminosilicate (fly ash) and the calcium silicate in the cement, raising the silicon concentration in the reaction medium and with it silicon uptake in both gels (Figure 6c).

At the same time, the Ca^2+^ and Al^3+^ ions present in the aqueous solution begin to diffuse through the hardened cementitious matrix. A small number of Ca^2+^ ions (not taken up in the C–S–H gel) interact with the N–A–S–H gel to form an (N,C)–A–S–H gel. Given the similar ionic radius and electronegative potential in Na^+^ and Ca^2+^, calcium replaces the sodium ions via ion exchange reminiscent of the mechanisms observed in clay and zeolites [61,72], maintaining the three-dimensional structure of the (N,C)–A–S–H gel [63]. Similarly, the C–S–H gel forming from the silicates in cement takes aluminum into its composition (preferably) in bridge positions [48,73,74], yielding C–(A)–S–H → C–A–S–H gels as the aluminum content rises (Figure 6d).

Where a sufficient store of the element is available, calcium continues to diffuse through the pores of the matrix and interact with the (N,C)–A–S–H gel. The polarizing effect of the Ca^2+^ (to form Si–O–Ca bonds) distorts the Si–O–Al bonds, inducing stress and ultimately rupture. At present, two hypothesis can explain the ion exchange mechanisms between the different gels produced in these type of cementitious systems: (a) the replacement of one Al^3+^ and one Na^+^ by two Ca^2+^ and (b) the replacement of two ions of Na^+^ by one Ca^2+^. As the N–A–S–H gel releases aluminum, less polymerized structures (C–A–S–H gels) will be formed. At the same time, that C–A–S–H gel formed in previous stages will incorporate more silicon and aluminum ions in bridging positions [75] (Figure 6e). However, we also have to consider that alkalis released to the pore solution might react with unreacted fly ash, then forming more N–A–S–H gel; and this last one can interact with C–S–H gel. In summary, an import part of the original alkalis can recycle and play an important role in the subsequent alkaline activation reactions. However, with time, these alkalis will become a part of the structure of the reaction products. It means that with time (with the reaction progress), the alkaline concentration will decrease until the equilibrium stage in pore solution is achieved. This process can be very slow in comparison with the very fast initial gel formation reactions. Currently authors are studying these systems by using different techniques (NMR, Electron Microscopy and Pores Solution Analysis) in order to confirm our hypothesis at long term (we are working with samples older than three years).

With time and under equilibrium conditions (attainable after longer reaction times), a (N)–C–A–S–H gel prevails. This is consistent with the behavior observed by the same authors in synthetic samples. 

In these complex cementitious blends, the products formed and their proportions depend on reaction conditions, including: the chemical composition, shape, mineralogy and particle size distribution of the prime materials (fly ash reactivity rises with its vitreous content [49] and with declining particle size [76]), as well as the alkalinity (pH) generated by the activator [20,22,50,64].

## 4. Effect of Alkaline Activator in Hybrid Alkaline Cement Hydration

Garcia-Lodeiro et al. [22], analyzing hybrid cements activated with solutions of different alkalinity, found that, while the type of alkaline activator impacted reaction kinetics and the formation of secondary reaction products (carbonates, AFm phases…) significantly, it did not appear to have any material effect on the main cementitious gels formed ((N,C)–A–S–H/C–A–S–H). The thermodynamically stable majority product was a mix of cementitious gels that formed irrespective of the activator used. Nonetheless, the relative amount of each gel was observed to depend on the activator. Whilst the presence of strong alkalis favored the dissolution of fly ash and the precipitation of an (N,C)–A–S–H gel, the use of more moderately alkaline compounds favored the formation of C–A–S–H gels [22].

### 4.1. Intense Activation

The vast majority of hybrid cement hydration studies have been conducted with activators (primarily NaOH or mixes of NaOH + WG) that render the medium highly alkaline [20,22,26,50,64]. The high alkalinity of these solutions (with pH values of over 13) favors speedy ash dissolution. Moreover, at ambient temperature fly ash activation is accelerated by the presence of Portland cement clinker. The explanation for this beneficial effect lies in the heat released during cement hydration, which would favor the chemical reactions inducing ash dissolution, setting and hardening.

Highly alkaline activators, such as NaOH, prompt the hydrolysis of Si–O and Al–O bonds (the OH^−^ ions act as catalysts), while the presence of soluble silica in the form of silicate ions enhances the polymerization rate of the ionic species present in the system [77,78]. Portland cement hydration, in turn, is affected by alkaline content (OH^−^ concentration) and the presence of soluble silica [68,69,79]. 

A number of authors have analyzed early age (1 to 28 days) hybrid cement behavior (MK + Ca(OH)_2_, BFS + OPC, FA + OPC...) when the material is hydrated in the presence of different concentrations of strong activators. They consistently observed that high alkalinity favors the formation of N–A–S–H/(N,C)–A–S–H gels to the detriment of C–S–H gels and inhibits portlandite formation. C–S–H gel formation is favored by milder alkalinity [18,22,26,56,57,58,59].

That the type of activator and the alkalinity generated affect reaction kinetics and the nature of the gels initially formed has been ratified by studies on later age cements [20,22]. As hydration progresses, only the most thermodynamically stable products are identified. In systems containing large proportions of fly ash and low proportions of Portland clinker the outcome is a mix of C–A–S–H and (N,C)–A–S–H gels [20]. The proportion of each gel depends on the alkaline activator, however. When a mild activator is used, C–A–S–H gels are generated, with a minority presence of (C,N)–A–S–H gels.

Some of the arguments against the use of pure alkali–activated cements have centered on practical health and safety issues around the preparation and storage of highly alkaline solutions on construction factory sites. Given that alkali–activated cements are often touted as low-CO_2_ alternatives to PC, the associated CO_2_ footprint of the alkali activators has come under scrutiny [80]. The manufacture of NaOH and Na_2_SiO_3_ are fairly energy intensive processes. As a general rule of thumb, Duxson et al. [81] contended that the CO_2_ footprint of manufactured NaOH and Na_2_SiO_3_ stands at around 1 t CO_2_/t. Furthermore, in hybrid alkaline systems, highly alkaline activators appear to adversely affect C–S–H gel stability, the kinetics of clinker calcium silicate hydration or both. The result has been a growing interest in ‘just add water’ hybrid alkaline cement formulations, in which milder alkalis are used.

### 4.2. Mild Activation

The mildly alkaline activators commonly used to hydrate these hybrid cements include weak (sodium carbonate) and strong (sodium sulfate) acidic salts (see Table 1). Whilst NaOH solutions generate pH values ranging from 13 to 14, the media containing these milder activators exhibit values from 7 to 13.

The effect of such soluble salts in raising the pH in the medium may possibly be attributed to their synergetic reaction with Ca(OH)_2_ [17,71,82], further to Equation (1):

*x*Ca(OH)_2_ + A*_x_*B*_y_* ↔ Ca*_x_*B_2_(s) + 2*x*A(OH)(ac) (1)

where *A* is an alkaline cation, normally Na^+^ or K^+^, and *B* is the anion in the respective inorganic salt. Alkalinity is raised provided that the anion in the inorganic salt used forms an insoluble calcium compound (Ca*_x_*B_2_), shifting equilibrium to the right in Equation (1).

The inorganic salts most widely used are listed in Table 1, along with their solubility products and the equilibrium calcium concentration. Lower calcium ion concentrations favor the precipitation of the inorganic calcium salt (Ca*_x_*B_2_) generated, shifting equilibrium further to the right and rendering the activator more effective.

Another advantage of inorganic salts over highly alkaline activators (such as NaOH and WG) is that they have a lower impact on the hydration reaction of the clinker component in hybrid cements. In these systems, the clinker must first react with water to generate portlandite (pH ~ 12.5), which then reacts with the inorganic salt to generate the respective insoluble calcium salt and Na^+^(OH)^−^: i.e., alkalinity is generated in situ, as indicated in Equation (1) (pH > 13). For instance, depending on whether the activator is a carbonate or a sulfate, its reaction with Ca(OH)_2_ yields calcium carbonate or hydrated calcium sulfate (possibly gypsum), as shown in Equations (2) and (3), as well as Na^+^(OH)^−^(aq.), thereby raising medium alkalinity. In addition, the heat released during initial OPC hydration favors and expedites the dissolution of supplementary cementitious materials at high pH [17,71,82].

Ca(OH)_2_ + Na_2_CO_3_ → CaCO_3_(s) + Na^+^OH^−^(aq.) (2)

Ca(OH)_2_ + Na_2_SO_4_ → CaSO_4_·2H_2_O(s) + Na^+^OH^−^(aq.) (3)

Another intrinsic factor in the use of inorganic salts as mildly alkaline activators is the role of the constituent anion, particularly in connection with the secondary phases precipitating in these systems. The use of Na_2_CO_3_ as an activator, for instance, has been shown to favor the formation of gaylussite-like carbonates and calcite at very early reaction times (2 h) [22]. In that study, gaylussite was shown to be metastable during alkaline fly ash cement hydration. The authors hypothesized that the temporary uptake of Na in precipitating gaylussite delays the alkali activation of glassy fly ash phases.

When in a subsequent study Na_2_SO_4_ was used to activate cements, no ettringite was observed at any of the ages analyzed [64]. As the OPC used in these systems is normally blended with gypsum, it should theoretically have been able to form ettringite. Even in studies on pure OPC, however, the presence of alkalis has been shown to prevent ettringite formation [68,69], favoring instead other C_3_A hydration products such as phase U [83,84]. Monocarboaluminate (Ca_4_Al_2_CO_3_·11H_2_O) has been observed to be the prevalent in seven-day product. Systems with carbonate-containing activators exhibit the same behavior [22]: No ettringite is detected, although AFm (hemi and monocarboaluminate) forms in the seven-day materials. 

Alahrache et al. [70] analyzed the effect of other, less conventional, mildly alkaline activators such as potassium citrate, sodium-potassium silicate and sodium oxalate on hydration kinetics and strength development in systems with high fly ash and low OPC contents. They found that the two latter activators were promising candidates for activation, for they shortened setting time and raised early age mechanical strength. The use of potassium citrate, however, retarded ash and clinker hydration, due either to the formation of complexes on the surfaces of these particles or to the hampering of C–A–S–H gel and AFm phase formation.

In a summary, the type of activator used has a direct impact on the secondary products precipitating and on reaction kinetics (essentially through the pH generated in the medium), accelerating or retarding the precipitation of the main reaction products. Irrespective of the type of activator used, however, the majority and most thermodynamically stable product in these non-equilibrium systems with limited amounts of calcium is a mix of cementitious gels. The proportion of each gel in the mix does, however, depend on the alkalinity generated in the medium.

## 5. Conclusions

This paper presents descriptive models for high fly ash-content hybrid alkaline cement hydration, a process that involves aspects typical of both PC and AAFA cement hydration. Hybrid binders arouse considerable interest given that their use would reduce CO_2_ emissions to much lower levels than traditional fly ash pozzolanic cements. 

In hybrid cements activated directly with highly alkaline chemicals (NaOH), the prevalence of N–A–S–H gel interferes with the normal hydration of the calcium silicate phases present in the clinker, thereby hindering C–S–H gel formation. Both Al and Na have been shown to favor the conversion of C–S–H gel to C–A–S–H-like gel. Conversely, the presence of Ca attributable to clinker hydration has been shown to modify the structure of the N–A–S–H gel formed during fly ash hydration, with the appearance of an (N,C)–A–S–H structure.

In hybrid blends activated indirectly with moderately alkaline compounds (such as alkaline sulphates, carbonates or phosphates), both N–A–S–H and C–S–H gels are observed to form at early ages. The initial rapid hydration of the calcium silicate present in the clinker generates sufficient Ca and alkalinity to convert part of the soluble alkaline salts to NaOH, which then activates the glassy phases in the fly ash, inducing paste setting. The role of the anion in soluble alkaline salt activators should not be underestimated. 

In hybrid alkaline cement systems, both N–A–S–H– and C–S–H gels have been proven to evolve toward C–A–S–H structures in older age specimens. Depending on the total amount of available Ca, a fraction of the N–A–S–H gel may remain in the system indefinitely.

## Figures and Tables

**Table 1:** Solubility (g of solute/100 g of water) and the Solubility Product Constants, K_sp_ of sodium and respective calcium salts and equilibrium calcium concentration (Equation (1)).

A = Anión	Na Salts		Ca Salts		
	Solubility g/100 g of Water Near to 25 °C	Ksp	Solubility g/100 g of Water Near to 25 °C	Ksp	(Ca^2+^)
OH^−^	100	110	0.160	5.5 × 10^−6^	11.1
F^−^	4.13	–	0.0016	5.3 × 10^−9^	1.1
CO_3_^2−^	30.7	1.2	0.00066	2.8 × 10^−9^	0.053
SO_4_^2−^	28.1	10.3	0.205	9.1 × 10^−6^	3.0
PO_4_^2−^	14.5	2.24	0.00012	2.0 × 10^−29^	0.019
